# Buffalo Medical Association

**Published:** 1859-12

**Authors:** W. H. Butler


ART. III.—*Proceedings of the Buffalo Medical Association—October Meeting.* Reported by W. H. Butler, M. D., Secretary.

Dr. Rochester reported the successful treatment of albuminuria, and dropsy following scarlet fever, by tannin. Had seen cases published in the "American Medical Monthly," and other periodicals, taken from Parisian journals, where this treatment had been successful. He had tried it in three instances, and succeeded in relieving anasarca by the tannin. The first two cases were children; both had convulsions and dropsy. Had first placed them on chlorate of potash and bark, then on iron, but with no decided advantage. The quantity given was three grains at a dose, dissolved in glycerine and water; the glycerine is an admirable solvent of the tannin. The urine in these cases was small in quantity, but contained a large amount of albumen.

Dr. Rochester also reported a case of hæmorrhage from the upper eyelid near the inner canthus of the eye, and from the umbilicus, of a new-born child. The child was born two weeks since. The parts of the mother were small, and it became necessary to use forceps. The child weighed eleven pounds; immediate examination showed the scalp and face to be free from any injury. The next day it was also examined, and found all right. On the third day his attention was called to the inner canthus of the eye; blood flowed from a small jet, falling over the cheek and nose. It came from under the brow, where it could not possibly have been reached by the forceps. Had first used compression, then a solution of alum, and the fourth day nitrate of silver. These were of no avail, and a solution of the perchloride of iron was used, and controlled the bleeding effectually.

Dr. Rochester reported a case of sudden death following a hysterical convulsion, giving the history of the case and the *post-mortem* appearances.

Dr. Wilcox remarked he had been present at the *post mortem* referred to, and had noticed the almost total want of muscular tissue; it seemed to have been substituted by fat. Thought the brain was the seat of the disease.

Dr. White presented a specimen of cauliflower excrescence attached to the lower segment of the uterus; being one of those spoken of at the last meeting as having been removed by the *ecraseur*. It had formerly been the practice of some surgeons to remove these with the knife, but the operation had latterly been abandoned on account of the danger of hæmorrhage. Of the two cases he had had during the last year, where he had removed the neck and lower part of the uterus by the *ecraseur*, both had completely recovered.

Some writers, in speaking of this instrument, had recommended tightening the chain and then resting occasionally; but Dr. W. did not think this necessary; he had not consumed more than five minutes after the chain was on the uterus before its removal, and had not in either case lost more than one or two ounces of blood. He had remarked that both patients were well; so they were, though the microscope showed the cancer cells, and it was too early to determine whether the disease would return; though in any case the patient would be more comfortable and have temporary relief by its removal.

Dr. White also reported a case of removal that afternoon (Oct. 4th), of a large fibrous tumor from the uterus, and presented the fresh specimen for the examination of members. In this case he had substituted a wire rope for the common chain in the *ecraseur*, and liked the substitute, as he was enabled by it to introduce the instrument well up in the vagina. The patient was 30 years of age, and of a very pale, bloodless complexion ; has lost a large amount of blood at different times.

Dr. Wilcox, whose patient it was Dr. White had removed the tumor from, remarked that he first saw the patient a year ago; was called on account of her flowing badly ; had been flowing for three weeks. He tamponned her, but it did not arrest it. Afterwards he cauterized with nitrate of silver two or three times. Had seen little of the patient until a day before the operation. He was called, and on examination he discovered the mass of fibrous matter which had that afternoon been removed by Dr. White.

Dr. Treat said the matter of fibro-cartilaginous tumors reminded him of a case where the patient had died after the removal, from pneumonia.

